# Minimal functional activity and presence of an antigen sink limit CD36 blockade as a macrophage-targeted cancer immunotherapy

**DOI:** 10.1371/journal.pone.0362100

**Published:** 2026-11-12

**Authors:** Elizabeth Kuczynski, Kyoko Nakamura, Gareth Browne, Andrea Gonzalez-Munoz, Francesca Delvecchio, Jane Coates-Ulrichsen, Mukul Tewary, Borjan Venovski, Jessica Dawson, Shreya Ahuja, Grace Opoku-Ansah, Amanda Watkins, Lorraine Irving, Tilbe Creigh-Pulatmen, Pablo Morentin Gutierrez, Jessie Salter, Rebecca Dudley, Mathilde Colombe, Allison Gerber, Kelli Ryan, Kathy Mulgrew, Sonja Hess, Matt Glover, Francisca Wollerton, Robert W. Wilkinson, Elina Timosenko, Alan Sandercock, Mark Cobbold, Simon J. Dovedi, Luca Melchiori

**Affiliations:** 1 Immuno-Oncology, Circuits-Engagers and Cell Therapy, Early Oncology R&D, AstraZeneca, Cambridge, United Kingdom; 2 Biologics Engineering, AstraZeneca, Cambridge, United Kingdom; 3 Dynamic Omics, CGR, Discovery Sciences, R&D, AstraZeneca, Gaithersburg, Maryland, United States of America; 4 Oncology Target Discovery, Chemistry and Drug Metabolism and Pharmacokinetics, Oncology R&D, AstraZeneca, Gaithersburg, Maryland, United States of America; 5 Immuno-Oncology, Circuits-Engagers and Cell Therapy, Early Oncology R&D, AstraZeneca, Gaithersburg, Maryland, United States of America; 6 BioPharmaceuticals R&D, AstraZeneca, Cambridge, United Kingdom; Showa University: Showa Daigaku, JAPAN

## Abstract

Targeting macrophages in the tumor microenvironment offers potential to relieve immune-suppression and improve anti-tumor immune responses. Unfortunately, a range of tumor-associated macrophage (TAM)-targeting agents in development has not yet translated into clinical benefit. We sought to identify novel targets on primary human macrophages through an unbiased phenotypic selection campaign. This work identified the scavenger receptor and lipid transporter CD36 as a potential target associated with anti-inflammatory phenotypes on macrophages. We first confirmed the ability of in-house generated and commercially available anti-CD36 monoclonal antibodies (mAbs) to inhibit CD36 binding and cellular uptake of oxidized lipids, both in cell-free settings and in engineered cell lines. However, we were unable to observe consistent or robust responses to CD36 blockade in primary human macrophages using a range of TAM-like and lipid-induced *in vitro* models. *In vivo*, CD36-KO mice showed reduced tumor growth, which implicated a CD36-dependent host impact on tumor progression. Importantly, anti-CD36 mAb failed to phenocopy this effect. Pharmacokinetic assessments revealed considerable target-mediated drug disposition with anti-CD36 mAb, limiting free plasma concentrations of the drug. A modified dosing regimen managed to increase drug concentration in plasma but still failed to elicit meaningful activity across primary tumor models, with/without anti-PD-L1. Although it remains clear that lipid uptake and metabolism is a key feature in TAM that can drive their immunosuppressive activities, our investigation raises concerns regarding the therapeutic targeting of CD36 due to redundancy in the lipid scavenging receptor family and a broad tissue expression profile. Together, these findings suggest that conventional monoclonal antibody blockade of CD36 has limited therapeutic potential in solid tumors, although outcomes may vary in different biological contexts.

## Introduction

Novel therapeutic approaches are required for cancer patients who do not achieve durable benefit with immune checkpoint inhibitors such as anti-PD-(L)1 [1]. Modulating the myeloid cell compartment within tumors represents a potential opportunity to enhance tumor-immune responses [2, 3]. Tumor-associated macrophages (TAM) are highly abundant in the tumor microenvironment (TME) and display a high degree of heterogeneity conferring both pro- and anti-tumor properties. Several attributes render these cells attractive targets for therapeutic intervention. First, TAM are associated with poor survival in many cancers [4] and with resistance to chemotherapy and immunotherapy [5–7]. Second, pro-tumorigenic TAM exert broad effects on the TME. For example, they reduce recruitment of cytotoxic T-cells and phagocytosis, express suppressive proteins and promote both angiogenesis and tumor invasion. Co-opting macrophages’ roles in innate and adaptive immunity could therefore lend broad, multicellular mechanisms of action of a potential therapeutic [8, 9]. Third, macrophages are functionally plastic. While older classifications describe TAM as either activated ‘M1-like’ anti-tumor TAM or alternatively activated ‘M2-like’ pro-tumorigenic TAM [10], they are now understood to adopt a spectrum of functional and phenotypic states in response to environmental cues [9]. Their cellular plasticity suggests it could be possible to ‘reprogram’ pro-tumor TAM to more anti-tumor states, while sparing beneficial TAM.

There has been intense activity in the development of myeloid-modulating cancer therapies, which has amounted, so far, to little clinical benefit. Over 200 TAM-reprogramming agents have been tested in over 700 clinical trials [3], the majority of which are biosimilars targeting a handful of molecules or pathways. These include molecules that stimulate TAM function (e.g., TLR agonists), block suppressive signalling (e.g., PI3Kγ, STAT3 inhibitors), modulate scavenger receptors (e.g., CD163), target phagocytosis or myeloid checkpoints (e.g., CD47-SIRPα, LILRB) or reduce suppressive metabolites (e.g., IDO1, arginase inhibitors). Unfortunately, strong clinical anti-tumor activity remains absent with these agents [3]; pathway redundancy and metabolic adaptation have been proposed as possible causes [2].

Phenotypic antibody selection, although not commonly used, is a discovery approach that can identify novel druggable targets [11]. Through phenotypic selections, panels of antibodies are isolated by phage selections against a relevant tissue or cell type, then screened for their ability to elicit a desired function in a target-agonistic manner [12]. To our knowledge, such an approach has not previously been applied to TAM, which are challenging to target due to the complexity of their behaviour [13]. Yet, a target-agnostic approach might uncover new ways to reprogram their activity.

Highly suppressive lipid-associated macrophages have been identified in human tumors [13–16]. This suppression is thought to be driven by a lipid-rich tumor interstitium [17–19], and extends to inhibition of T-cell function and infiltration. Indeed, lipid droplet-dependent fatty acid metabolism has been implicated in shaping the immunosuppressive phenotype of TAMs [20]*.* It is not clear whether any upstream pathways, such as lipid uptake, are actual drivers or merely associated factors of immune suppression in these cells, and whether a drug can revert this phenotype.

Here we report our campaign to discover novel targets on TAM through phenotypical selection, which identified the lipid transporter and scavenger receptor CD36 as a potential novel target. CD36 is a multi-functional transmembrane receptor with a wide range of ligands, including TSP-1, oxidized phospholipids and low-density lipoprotein (LDL), hexarelin, beta-amyloid, long-chain fatty acids (LCFA), *Plasmodium falciparum*-infected red blood cells (RBCs), bacterial cell wall components, microparticles and apoptotic cells with a range of binding sites on CD36 [21]. Additionally, CD36 is a lipid transporter for LCFA and oxidized lipids [22]. Our subsequent *in vitro* and *in vivo* investigations to validate the target’s functionality and tractability confirmed that mAb-mediated inhibition of CD36 could block lipid uptake via this receptor. However, we did not observe robust functional effects using *in vitro* macrophage culture models. Moreover, we identified the existence of a substantial antigen sink for CD36, rendering systemic administration and inhibition of CD36 impractical. This work highlights that even with the use of mAbs that are high-affinity and selective for CD36 inhibition, pathway redundancy for lipid sequestration and widespread expression of the target on other cell types limits the therapeutic potential of targeting CD36.

## Materials and methods

### Cell selections

Healthy donor leukapheresis samples were obtained from the UK National Health Service Blood and Transfusion services (Addenbrooke’s Hospital, Cambridge). Peripheral blood mononuclear cells (PBMC) were isolated by Ficoll-Paque centrifugation (GE Healthcare). Monocytes were negatively selected with the EasySep human monocyte enrichment kit without CD16 depletion (StemCell Technologies) using the Robosep (StemCell Technologies). The monocytes from 6 donors were aliquoted and frozen.

For the panning selections, approximately 10e6 viable monocytes per donor were defrosted and pooled. The cells were cultured at a density of approximately 15e6 cells per T175 flask (Corning) with RPMI-1640 media (Sigma) plus 10% FBS and 1% Pen/Strep supplemented with 100 ng/mL macrophage-colony-stimulating factor (M-CSF; Peprotech) for 7 days to allow differentiation. At day 7, the cell subpopulations were generated adding only media (M0), or polarising with interferon-gamma (IFN-γ; 20 ng/mL R&D systems) for M1-like cells or IL-4 (100 ng/mL, R&D systems) for M2-like macrophages. At day 8, flasks containing M0, M1- and M2-like cells were activated with 20 ng/mL lipopolysaccharide (LPS-SM Ultrapure, Invivogen) for 24 h.

The cells from individual and pooled donors passed the ELISA (Duoset R&D systems) quality control in which the release of IL10, IL12, CXCL11 and CXCL13 were measured in the supernatants.

### Phage Display with 4 macrophage subpopulations (M0, M0 + LPS, M1 + LPS, M2 + LPS)

A single-chain variable fragment (scFv) synthetic library (Restricted Framework (REF) library) made in-house was used for cell selections. Briefly, the REF library was built recombining a heavy chain (IGVH1–69/DP-10) with a light chain (IGVL1–44) in which the CDR3s were randomized. The REF library aliquots were first blocked in media (RPMI-1640 + 10%FBS) and then deselected against a set of commonly expressed macrophage proteins (HLA-DR (Abcam); IL-1R β, IL-2 Rα, β, γ, IL-4 R, IFN-γR1, GM-CSFα, β, CD11b,c, CD14, CD16a,b, CD32 a,b/c, CD33, CD40, CD45, CD64, CD80, CD86, CD115, TLR2, TLR4 (R&DSystems).

The cells in the flasks were washed with cold media and then incubated on ice with 5 mL of cold media supplemented with 10 μM of Cytochalasin D (Sigma) for 20 min. The blocked and deselected phage were added to the cells and the flasks incubated for 1 hr at room temperature on a shaker. After washing 4 times with 5 mL cold PBS +Cytochalasin D, the phage were eluted off using 2.5 mL 250 μg/mL trypsin (1:10 dilution; Sigma) in 100 mM sodium phosphate, pH 7, and incubated for 37°C for 15 min. To neutralize the trypsin, 1 mL of media was added, cells were scraped and supernatant containing the eluted phage collected. TG1 cells were infected with the eluted phage and then plated in 2x TYAG (100 μg/mL ampicillin, 2% glucose) bioassay plates. The second and third round of selections were performed as the first one except that round 3 was not deselected for known macrophage antigens.

### Reformatting the scFv to human IgG1 TM

The round 2 and 3 outputs were population-subcloned from the pCANTAB6 scFv format into human IgG1 TM (triple mutation in Fc to minimize interaction with FcR) in the mammalian IgG expression vector pmIgG for the purpose of high-throughput screening in IgG format and subsequently expressed in a suspension Chinese hamster ovary (CHO [23]) cell line by transient transfection. Antibodies were purified by protein A-based capture.

### Macrophage Binding Assay - mirrorball^TM^

The binding profile of these clones to macrophage subpopulations was initially determined using a high throughput mirrorball^TM^ (TTP LabTech) cell binding assay– a fluorescence-based wash assay performed at 4°C in the presence of Cytochalasin D. The four macrophage subpopulations were generated and plated in 96 well clear bottom plate TC-treated, flat bottom with lid (ThermoFisher Scientific). The control antibodies, NIP-228, anti-TLR2 and anti-CD32 were made in house as human IgG1 TM. Cells were incubated with primary antibody for 1 h at 4°C and washed with assay buffer. As secondary Ab, Alexa Fluor 647-conjugated AffiniPure F(ab’)2 fragment goat anti-human IgG, Fcγ Fragment specific (Jackson ImmunoResearch Lab Inc.) was used at 1 μg/mL 1/1000 in assay buffer (0.1% BSA (Sigma) in Hank’s Balanced Salt Solution (HBSS; Sigma). Cells were incubated with secondary for 1 h at 4°C and washed with assay buffer. Fluorescent binding was detected using a mirrorball^TM^ microplate cytometer system (TTP LabTech). This assay allowed us to acquire 879 hits, some of which were macrophage subpopulation-specific, while the majority bound to multiple macrophage subpopulations.

### High-Content Imaging screening on primary human macrophages

Macrophages stained for the mirrorball^TM^ assay were washed, fixed with 4% p-formaldehyde and stained with CellMask dye (green) and Hoechst3342 nuclei dye (blue). Image acquisition was performed with ImageXpress (Molecular Devices) with filters, and exposure times according to the manufacturer’s instructions. Images were quantified using Columbus Analysis System (PerkinElmer). From a total of 879 IgG, 402 were selected based on cell-associated binding. These clones were selected for functional screening.

### Target Identification – membrane protein binding screen

Target identification was performed using a membrane protein binding screen; briefly, an array of ~2900 plasmid vectors, comprising human cell surface protein cDNA sequences downstream of a CMV promoter, were combined at 30 ng/plasmid with Lipofectamine LTX (1:200 dilution) transfection reagent in a total volume of 20 μL OptiMEM media in 384w clear-bottomed PDL-coated plates (Greiner), followed by 293FT cells at 4000 cells/well in 30 μL OptiMEM media. Each plate included a positive control well encoding an Fc-gamma receptor to detect antibody binding via the Fc region. The cells were cultured for 2 days at 37°C / 5% CO_2_. Antibodies were combined with anti-human-IgG-AF647, then added to the cells and incubated for 3–4 h prior to measuring bound fluorescent signal using a mirrorball^TM^ plate cytometer. Wells showing positive binding signal were identified (ignoring several Fc-receptors present in the array), and the vectors transfected in these wells were prepared from original glycerol stocks, and sequence-confirmed by Sanger sequencing. A smaller scale repeat of the transfection array was then performed in a similar set-up, with the sequence-confirmed plasmids and a larger panel of antibodies from the functional screening hits described above.

### In silico analysis of CD36 expression on macrophages in cancer

In silico analyses were performed using Bioturing. The plots were created with Vinci software (BioTuring Inc., San Diego, CA, USA).

### Biolayer Interferometry

Antigen-binding assays were performed by biolayer interferometry on the Octet RH96 (Sartorius) at 25°C in assay buffer containing PBS (14190–169, ThermoFisher Scientific), 0.1% [volume for volume (v/v)] BSA (A9576-50ML, Sigma), pH 7.4, using tilted-bottom, black 384-well plates (18–5080; Sartorius). Assays to test the affinity of IgGs for human CD36 were set up using OCTET anti-human IgG Fc capture (AHC) biosensors (18–5060; Sartorius) according to the manufacturer’s instructions. Following 5 minutes equilibration in assay buffer the antibodies (anti-CD36 IgG1 or isotype negative control) were loaded onto the AHC biosensors at 5 μg/mL for 1 min for a wavelength shift of approximately 0.7 nm, followed by 1 min dissociation in a fresh well containing buffer only. The biosensors were then moved to wells containing a titration curve of human CD36 avi-HIS (1.56 to 100 nM) or buffer only, and the wavelength shift was measured over 5 min. Finally, the biosensors were moved to fresh assay buffer wells for a 10 min dissociation. Data were analysed with Octet Analysis Studio, version 12.2 (Sartorius) to calculate the K_D_, k_on_ & k_off_ over the association/dissociation steps following reference sensor subtraction. For oxLDL binding inhibition studies, assays were set up with High Precision Streptavidin (SAX2) Biosensors (18–5137; Sartorius) according to the manufacturer’s instructions. Following 5 min equilibration in assay buffer the first antigen, biotinylated recombinant human CD36 (CD6-H82E9, Acro Biosystems), was loaded onto SAX2 biosensors at 5 μg/mL for 1 min for a wavelength shift of approximately 0.7 nm, followed by 1 min dissociation in a fresh well containing buffer only. The biosensors were then moved to a well containing the test antibodies (anti-CD36h/m IgG1 or isotype negative control) at a concentration of 25 μg/mL, and the wavelength shift was measured over 5 min. After a 1 min dissociation period in fresh assay buffer, the biosensors were moved to wells containing the oxLDL (L34357, ThermoFisher Scientific) at 5 μg/mL for a further 5 min, and the wavelength shift was measured. Finally, the biosensor was moved to a fresh assay buffer well for a 5-minute dissociation period. Data were analysed with Octet Analysis Studio, version 12.2 (Sartorius) to display the binding curves over the full binding time course or the oxLDL association/dissociation stages only. This binding curve data was exported as a  .csv file & plotted in GraphPad Prism.

### Homogeneous Time-Resolved Fluorescence (HTRF) Epitope competition assay for human CD36 binding

The anti-CD36 antibodies, in hIgG1 TM or mIgG1 D265A format and the relevant isotype negative controls, were screened in an epitope competition HTRF® assay for inhibition of Dylight 650 (84536, ThermoFisher Scientific) labelled Macro0168 hIgG1 TM binding to biotinylated human CD36 (CD6-H82E9, Acro Biosystems). The test IgGs were titrated in duplicate assay buffer containing PBS (14190−169, ThermoFisher Scientific), 0.1% [volume for volume (v/v)] BSA (A9576-50ML, Sigma), pH 7.4 & 10 μL added to each well of a 384-shallow well assay plate (4513, Corning). Dy650-Macro0168 hIgG1TM was diluted 20 nM in PBS (14190−169, ThermoFisher Scientific), 0.1% [volume for volume (v/v)] BSA (A9576-50ML, Sigma), 0.4 M Potassium Fluoride (26820.236, VWR), pH 7.4 & 5 μL added to each well of the assay plate. This was followed by addition of 5 μL of the preincubated 20 nM biotinylated human CD36 & 7.3 nM Streptavidin-Eu cryptate (610SAKLB, Revvity) mix to each well on the assay plate. Assay plates were incubated for 3 h at room temperature before the reading of time-resolved fluorescence at 620 nm excitation and 665 nm emission using an EnVision plate reader (Revvity). Data were analysed by calculating percent ΔF and percent specific binding & plotted in GraphPad Prism.

### CHO DiI-oxLDL uptake assay

CHO-K1 cells (parental, and two clonal lines engineered to overexpress human and mouse CD36) were cultured using F12 + 10% FBS (16000044, Gibco) for routine maintenance and passaged at a ratio of 1:25 every three days or at ~80% confluence. Two days prior to starting the assay, 20 μL of a 200K cells/mL cell suspension was seeded per well of a Collagen-coated glass-bottom 384-well plate (781956, Greiner) and incubated at 37°C & 5% CO_2_ for 24 h. Next day media was replaced with 20 µL of CD-CHO medium (10743029, ThermoFisher Scientific) and cells incubated overnight. 20μL from each well of the antibody dilution series plate was transferred into the assay plate and the plates were incubated at 37°C for 45 min. 20μL of 50 µg/mL DiI-conjugated oxLDL (L34358, ThermoFisher Scientific) was added to each well. Plates were incubated at 37°C for 6 h, then washed three times with PBS prior to fixation with 4% Paraformaldehyde (28906, ThermoFisher Scientific) for 15 min. The fixed plates were stained with cell mask deep red (C10046, ThermoFisher) and Hoechst (62249, ThermoFisher Scientific) and imaged in the ImageXpress Micro Confocal HT.ai High-Content Imaging System (Molecular Devices) using a 10X objective. The images were analysed using Columbus Image Analysis System (Revvity) for Total DiI-oxLDL Spot Area and the data were plotted in GraphPad Prism. CD36 expression was confirmed by flow cytometry (S1B Fig) using Macro0579 primary antibody for detection of CD36 and Alexa Fluor® 647 AffiniPure Goat Anti-Mouse IgG Fcγ (115-605-164, Jackson ImmunoResearch) as secondary antibody for detection.

### Primary human macrophage cultures

Human PBMC were isolated from healthy human donor blood cones by generating a Ficoll gradient (17-1440-02, GE Healthcare). Human CD14 + monocytes were isolated by positive selection using human monocyte enrichment kit without CD16 depletion (19058, Stem Cell Technologies). Monocytes were differentiated to macrophages in the presence of 50 ng/mL M-CSF (435435, Peprotech) in flasks containing media (RPMI 1640 GlutaMax media (61879−010, Gibco), 10% heat-inactivated FCS (10082147, ThermoFisher Scientific), 1% Penicillin Streptomycin (15140−122, Gibco)). Cells were supplemented with fresh M-CSF added midway during differentiation by adding it on top of existing media. Macrophages were harvested using Accutase (6964, Sigma) after 6 or 7 days and seeded into 96 well flat-bottom plates for assays (3598, Corning). Macrophages differentiated with M-CSF only are termed ‘M0’ or ‘unpolarized’ macrophages. All cell incubations were performed in a humidified incubator set at 37°C and 5% CO_2_.

### TAM assay

MDA-MB-231 tumor cells were cultured in regular media for 3 days until ~70% confluency in 0.1% FCS, 1% Pen Strep, RPMI media for 3 days. The tumor cell line supernatants were collected, and FCS in the media topped up to 10% and aliquots frozen.

Tumor conditioned media (TCM) was prepared 1:1 with regular RPMI media containing final M-CSF 50 ng/mL. Tumor associated macrophages (TAM) were cultured at 1.36e6 cells/mL in 25 mL tumor conditioned media in T175 cell culture flasks (Corning). An additional 25 mL of this media was added to cells mid-way through culture. Day 6 or 7, TAM and M0 were harvested. Cells were seeded at 35,000 cells/well and treatment with anti-CD36 antibody (Macro0574) was added at 7.5 μg/mL. Cells were incubated for 48 h. Additional LPS-treated controls were stimulated a further 24h (2 ng/mL). RNA was harvested as outlined below.

### IL-4 polarized macrophages

Differentiated primary human macrophages were seeded into flat-bottom 96-well plates at a density of 35,000 cells/well and allowed to rest 24 h in regular media. On day 8, media was changed to 0.1% FCS containing media for 5 h. Then media was replaced with 10% FCS containing media for CD36 pre-blocking at 1.5X concentration (10 μg/mL for hIgG1TM isotype control, Macro0581 or Macro0568. 30 min later, macrophages cultures were topped up with regular media or 20 ng/mL final concentration IL-4 (200−04, Peprotech). Cells were cultured 24 h prior to harvest for RNA and Fluidigm analysis. In a parallel set of samples, supernatants were collected from unpolarized or IL-4 polarized macrophages after a further 24h re-stimulation with or without LPS (2 ng/mL). TNFα levels were evaluated by ELISA (DY210, R&D Systems) following the manufacturer’s protocol.

### Gene expression analysis

RNA was isolated using the MagMax RNA Isolation kit (AM1830, ThermoFisher Scientific) using the KingFisherFlex (ThermoFisher Scientific). For TAM experiments, cDNA was synthesized using the High-Capacity cDNA Reverse Transcription Kit (4368813, ThermoFisher Scientific). Quantitative PCR (qPCR) was performed using Taqman fast advanced master mix (4444964, ThermoFisher Scientific), with target genes on FAM/MGB and the 18S housekeeper gene in multiplex (see S2 Table for all assay details) on the QuantStudio 12K Flex (Applied Biosystems). For experiments with IL-4 as polarizing stimulus, high-throughput qPCR was performed using Standard Biotools Dynamic Array 96x96 IFC. cDNA was synthesized and target genes pre-amplified for a total of 16 cycles according to manufacturer’s protocols. qPCR was performed on the Biomark X (Standard Biotools). For high-throughput qPCR experiments, the average of housekeeping genes *HPRT1* and *GAPDH* was selected as internal reference control genes due to their stability across stimulation conditions. The delta-delta Ct method was used to quantify target gene expression, and fold-change calculated using the calculation 2^-ddCT.

### Primary human macrophage polarization/stimulation assays

Monocytes were differentiated in the presence of M-CSF media (M0 differentiation) supplemented with oxLDL (L34357, ThermoFisher Scientific) in the presence or absence of antibody treatments, where indicated. For macrophage phenotyping experiments by flow cytometry, monocytes were seeded at 50,000 cells/well in 96-well assay plates and treated with M-CSF media containing either 5 or 50 μg/mL oxLDL or PBS vehicle control. Concurrently, 10 μg/mL of mouse IgG1, or CD36-blocking antibodies (Macro0574 or Macro0579) were added then assayed for surface protein markers (see S2 Table for a list of staining reagents).

To assay cytokine production following oxLDL, monocytes were differentiated in flasks in the presence of M-CSF, 50 μg/mL oxLDL or PBS and treated with 5 μg/mL of either mIgG1 isotype control, Macro0579 or Macro0574. Treated-oxLDL or M0 macrophages were re-seeded into 96-well assay plates and stimulated with 2 ng/mL LPS for 24 h. Conditioned media were collected and assayed for TNFα levels by ELISA (R&D Systems).

### Mass spectrometry-based proteomic evaluation of stimulated macrophages

Monocytes isolated from four donors were seeded in cell culture dishes (10062−880, VWR) and treated with or without oxLDL (50 μg/mL) and single- or combination- of 1 μg/mL Macro0579 anti-CD36 antibody, anti-CD204 antibody or mouse IgG1 isotype control antibody. On day 6, the cells were harvested, and the resulting pellets were lysed using ultrasonication in 5% SDS lysis buffer supplemented with Halt™ protease and phosphatase inhibitor (ThermoFisher Scientific). Next, 25 µg protein extracts were reduced and alkylated in the presence of 40 mM Chloroacetamide (CAA), 10 mM Tris(2-carboxyethyl) phosphine (TCEP), and 100 mM HEPES buffer, pH 8 for 1.5 hours at 95°C. Samples were mixed with a 1:1 blend of hydrophilic and hydrophobic carboxylate beads (Cytiva) at a 10:1 bead/protein ratio. Using an automated SP3 workflow on a Kingfisher Flex system (ThermoFisher Scientific), bead-bound proteins were washed twice with 70% ethanol and once with 100% acetonitrile, then digested with Trypsin/LysC mix (Promega) at 1:25 enzyme/protein ratio overnight on a shaker at 37°C. The resulting peptides were desalted using the Oasis µElution plate (Waters), dried under vacuum, and reconstituted in 0.15% formic acid.

The samples were analysed using a timsTOF Pro2 mass spectrometer (Bruker Daltonics) connected to an Evosep One (Evosep). To ensure reliable identification, all data were acquired in duplicate. For LC separation, 200 ng peptide mixture was loaded onto a PepSep™ C18 column (8 cm length, 150 µm inner diameter, 1.5 µm particle size; Bruker Daltonics) which was maintained at 50°C. Peptides were separated at a steady flow rate of 1 µL/min using the 60 samples per day (SPD) method. Data was acquired in positive ion dia-PASEF mode (as described by Meier et al. 2020), with a TIMS accumulation time of 100 ms. Both MS1 and MS2 scans were recorded between 100–1700 m/z range, with ion mobility resolution set to 0.70 and 1.3 V-s/cm^2^, over a ramp time of 100 ms. Using dynamically ramped collision energies ranging from 20 eV at 0.6 1/K₀ to 52 eV at 1.6 1/K₀, MS2 fragmentation was conducted across 24 ion mobility windows, each with a 25 m/z isolation width, completing a cycle time of 1.38 seconds.

All DIA raw files were analyzed using DIANN (v1.8.1) integrated into an in-house cloud-based quantms pipeline (Dai et al., 2024). For protein identification, MS/MS spectra were searched against the UniprotKB SwissProt human database using default settings for fully tryptic peptides with up to 2 missed cleavages, a minimum of 6 and maximum of 40 amino acids. Carbamidomethylation (C, + 57.0215 Da) was the fixed modification, with oxidation (M, + 15.999 Da) and protein N-terminal acetylation (+42.0106 Da) set as variable modifications. Peptides and proteins were filtered at a 1% False Discovery Rate (FDR), with DIA-NN automatically determining MS1 and MS2 mass tolerances. The MaxLFQ algorithm (Cox et al., 2014) within DIA-NN was used for protein quantification with retention time-based cross-run normalization enabled. Differential abundance analysis between different treatment conditions was performed using MSstats (v.4.1.0) (Choi et al., 2014) which utilized normalized protein quantities averaged from two technical replicates. Protein ratios with a 0.5-fold change and a p-value of <0.05 were classified as differentially expressed. Functional annotations and pathway enrichment of differentially regulated proteins was evaluated using GeneOntology terms and the Ingenuity Pathway Analysis (IPA, Qiagen) software.

### Flow cytometry oxLDL uptake assay

Monocytes were differentiated into macrophages by treatment with M-CSF for 6–7 days. Cells were incubated with CD36 blockade antibody or sulfo-N-succinimidyl oleate (SSO, ab145039 Abcam) in serum free medium for 2 h. DiI-oxLDL (20 μg/mL; L34358, ThermoFisher Scientific) was added and incubated for another 4 h. Cells were detached by treatment with cell dissociation buffer (252549, Sigma) and cells were gently scraped using pipette tips. Cells were further stained with live/dead blue (L34962, ThermoFisher Scientific) then DiI fluorescence level was detected by BD Fortessa and analysed on FlowJo.

### In vivo studies

In vivo studies were performed using eight- to ten-week-old female C57BL/6 (JAX:000664) mice or B6.129S1-Cd36tm1Mfe/J CD36-knockout (JAX:019006; CD36-KO) mice obtained from Jackson laboratory. The CD36-KO mice carry a neomycin cassette in opposite transcription orientation in exon 3 of CD36 gene that leads to its silencing [24]. Mice were housed in AstraZeneca vivarium, accredited to Association for Animal Accreditation of Laboratory Animal Care, with free access to food and water *ad libitum* and were cared for daily by trained personnel. Mice were acclimatized to the vivarium conditions for a week and handled according to the Home Office Animals Scientific Procedures Act, 1986, UK. All animal work was carried out under a Home Office approved project licence (PPL5259481) as well as according to the institutional ethical guidelines and protocols.

Humane endpoints were utilized for all in vivo experiments. Mice were humanely killed immediately by cervical dislocation and secondary confirmation of death if any of the following occurred: tumor volume reached 10% of body weight; tumors developed a lesion that is either weeping, holed, necrotic, or a deep pinhole lesion; body weight loss reached 20% compared with maximum weight. In addition, mice were humanely killed if they had combined clinical signs of distress (e.g., intermittent hunched posture, anorexia, dehydration, marked piloerection, and lethargy) that could not be alleviated within 48 h. The duration of the experiment is shown in the figure. Animal health was monitored at least once daily by trained personnel. All regulated procedures were performed by qualified Home Office Personal license holders. Anesthesia was induced by inhalation of isoflurane (1–5%) and maintained under 1–2.5% isoflurane prior to tumor implantation and terminal cardiac blood sampling. No mice died prior to meeting criteria for euthanasia.

Mice were subcutaneously injected with 100 µL of 5e3 B16F10 cells or 5e5 MCA205 or MC38 cells or 1e5 4T1 cells (intra-mammary fat pat) in 50% growth factor-reduced Matrigel (Corning) and dosed intraperitoneally with either anti-CD36 (Macro0579) or anti-PD-L1 antibody (clone 80) mIgG1 (AstraZeneca) or with an isotype control antibody on the day of tumor cell implantation or starting after 7 days. Tumor growth was monitored three times a week using calliper measurements. For CD36 expression assessments, mice were euthanized 14 days post-implantation and tumors digested using a buffer of collagenase, DNase and hyaluronidase and then mechanically dissociated on the GentleMACS. Cells passed through a 70 μm cell strainer then stained for viability (LD aqua, ThermoFisher Scientific), and surface markers CD45 (clone 30-F11, BV785, BioLegend), CD11b (clone M1/70, BUV395, BD Bioscience) and CD36 or its isotype control (clone HM36 for CD36, APC, ThermoFisher Scientific). Samples were acquired on a BD Fortessa and analysed on FlowJo. A CD36 binding antibody clone HM36 (eBioscience) was evaluated for competition against Macro0579 by incubating 1e6 CHO-K1 mCD36 cl.14 cells (1E6 cells/mL assay concentration) with a titration of unlabelled competing antibodies. The plates were incubated on ice for 3 h before reading on the iQue3. Tumors from B16F10–implanted mice treated with 20 mg/kg Macro0579 five times per week were dissociated into single-cell suspensions, stained with myeloid markers and a competing anti-CD36 antibody clone (HM36), and analysed by flow cytometry. CD11b⁺CD19 ⁻ myeloid cells were gated, and CD36 expression and target engagement were assessed by median fluorescence intensity (MFI) using FlowJo v10.8.1, comparing Macro0579- and isotype control-treated groups.

Tumor volume group comparisons were done on data from a single timepoint using either ordinary or non-parametric ANOVA as indicated in the figure legend, followed by pair-wise multiple comparisons tests if the ANOVA p < 0.05. The appropriate multiple-comparisons test was chosen after a test for normality.

### Pharmacokinetic (PK) evaluation of anti-CD36 (Macro0579)

The PK of the anti-mouse CD36 antibody in plasma was evaluated by administering the antibody at doses of 10 or 20 mg/kg to mice via IP in either single-dose or repeat dosing studies. The drug solution consisted of antibody in phosphate-buffered saline (PBS) and was administered at a volume of 10 mL/kg. At defined time points after injection, blood was collected from the tail vein without anaesthesia and mixed with lithium/heparin. A terminal cardiac bleed was performed and blood was collected in tubes mixed with lithium/heparin. Plasma was obtained by centrifuging the blood (10,000 g, room temperature, 5 min).

In single-dose studies, wildtype C57bl/6 or CD36-KO mice were administered a single 10 mg/kg IP dose of Macro0579 and blood was collected at defined intervals to assess anti-CD36 binding to platelets and RBCs or for plasma assessments of free (unbound) Macro0579. Cells from whole blood or platelet isolations were stained with anti-mouse IgG1 A647-conjugated secondary antibody (BioLegend) for 20 min at room temperature, washed in FACS buffer, then stained for RBC (Ter119-PeDazzle, BioLegend) and platelet markers (CD150-BV421, CD41-AF488, BioLegend). Samples were acquired fresh on a BD Fortessa.

### Anti-CD36 antibody measurement in plasma

Frozen plasma aliquots were assessed for free concentrations of unbound Macro0579 using an in-house developed ELISA. Briefly, high-binding 96-well plates (44-2404-21, Nunc) were pre-coated overnight with 2 µg/mL streptavidin (434301, Invitrogen) diluted in PBS, washed (3x with PBS-0.5% Tween-20), blocked 1 h with 1% BSA (A6003, Sigma) in PBS, washed, and incubated for 1 h with biotin-hCD36 (CD6-H82E9, Acro Biosystems) at 0.5 µg/mL in 1% BSA/PBS. Samples and standards were prepared in duplicate or triplicate and incubated 2 h diluted in 1% BSA/PBS or in matrix buffer (1% BSA with matched dilution of WT C57bl/6 mouse plasma). Plates were washed, incubated for 1 h with anti-mouse IgG conjugated to HRP (1:2,500 dilution, A2554, Sigma). After a final wash, the degree of mouse IgG (Macro0579) bound to immobilized CD36 was detected using QuantaBlu Fluorogenic Peroxidase kit (14169, ThermoFisher Scientific) on an Envision plate reader (excitation 315nm, emission 420 nm; (Revvity). The dynamic range for detection was approximately 1–250 ng/mL Macro0579. The minimum required dilution where there is minimal interference in the assay was determined to be 1:25. All incubations were performed at room temperature with gentle shaking.

### Human biological samples

All samples were obtained with written informed consent from anonymized patients. Studies were conducted in accordance with recognized ethical guidelines (Declaration of Helsinki) and were approved by the East of England – Cambridge East Research Ethics Committee (HTA license number 12109).

## Results

### Phenotypic Selections revealing CD36 as a target on TAM

With the aim of identifying novel targets for reprogramming TAM, we embarked upon a target-agnostic phenotypic screening campaign using phage display selections performed on primary human macrophages. Most TAM are thought to originate from circulating monocytes [10]. Monocytes from healthy donor PBMC were differentiated with M-CSF to macrophages and then polarized *in vitro* as follows*:* unpolarized (M0), LPS stimulated (M0 + LPS), IFN-γ + LPS stimulated (M1-LPS) and IL-4 + LPS stimulated (M2-LPS). This approach was selected to capture extreme macrophage polarization states [9], and thus increased the range of potential biological responses and targets available for binding.

After two rounds of panning selections on adherent macrophage populations, the phage outputs were re-cloned in bulk from scFv into the effector-function attenuated hIgG1-L234F/L235E/P331S triple mutant (TM) format [25] and then expressed in high throughput in mammalian cells (Fig 1A). More than a thousand unique IgG clones were then tested in a high throughput cell binding assay, identifying a panel of 879 IgGs as binding hits. Most of the antibodies (72%) at this stage were cross-reactive to macrophage populations. 22% were M0 macrophage specific. A minority were specific to either LPS-activated M0, M1 and M2 macrophages (1.5%, 1.5% and 2.6%, respectively).

**Fig 1:**
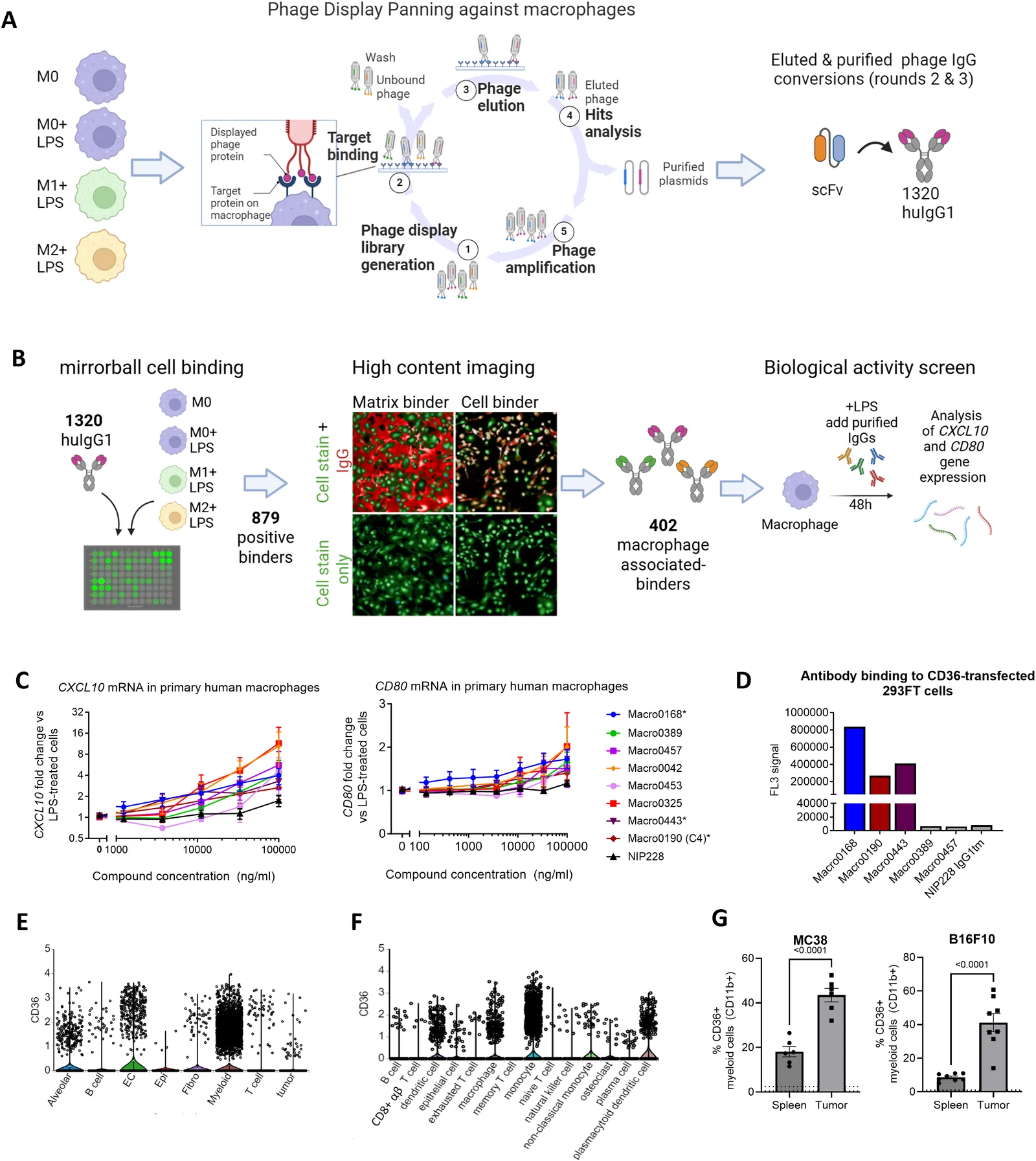
Agnostic identification of CD36 as a potential target on tumor-infiltrated macrophages. A. Cell phenotypic selections for IgGs targeting suppressive macrophages were performed on human macrophages (unstimulated M0, LPS stimulated, IFN-γ + LPS stimulated (M1), or IL-4 + LPS stimulated (M2)) using a synthetic scFv library (REF library). After two rounds of selections, the scFv from the eluted phage were converted into huIgG1TM. **B.** A high-throughput binding screening was performed on each of the induced macrophage populations by mirrorball^TM^ and high-content imaging. Two functional screens measured the expression of *CXCL10* and *CD80* genes in LPS-stimulated macrophages treated with mAbs for 48h. **C.** Expression of *CXCL10* or *CD80* normalized to *GAPDH* housekeeper gene was quantified in 6 human donors. **D.** Three functional hits demonstrating binding to CD36-transfected 293FT cells as assessed by mirrorball^TM^. **E.** Log-normalized CD36 expression from single-cell RNAseq analysis of human NSCLC (n = 5) [26] and **(F)** melanoma patients (n = 25) [27]. Whiskers represent the min-max values. **G.** CD36 expression on tumor- vs spleen-infiltrated CD11b+ myeloid cells in MC38 (n = 6) and B16F10 (n = 8) subcutaneous tumor-bearing mice, as assessed by flow cytometry. Tumors were analysed 14 days post-implantation of tumor cells and expression was compared to isotype control antibody (dashed lines). P-value represents an unpaired T-test result.

High-content imaging identified that a subset of the antibodies bound to the extracellular matrix secreted by the macrophage cultures, reducing the number of direct cell-binding IgGs to 402 (Fig 1B). 352 macrophage-binding antibodies were then screened functionally for their ability to reprogram primary human macrophages into an immunostimulatory phenotype of *CXCL10* and *CD80* gene induction. Eight reproducible hits, particularly the clone termed Macro0168, induced M1 markers *CXCL10* (a pro-inflammatory chemokine) and *CD80* (a co-stimulatory receptor) on LPS-stimulated primary human macrophages in several donors (Fig 1C). To identify the antigens recognized by these functionally active antibodies, we used an arrayed cDNA library of human membrane proteins transfected into HEK293 cells [28]. This approach demonstrated that at least 3 out of the functional hits specifically bound to cells overexpressing CD36 (Fig 1D). All hits were cross-reactive against M0, M1 and M2 macrophages populations in the selection screen.

CD36 is a driver of pathogenic foamy macrophages in atherosclerosis through oxidized low-density lipoprotein (oxLDL) uptake [29, 30], involved in alternative M2 activation of macrophages [31, 32]. Its lipid uptake function is linked to suppressive tumor-associated myeloid cells [14, 15]. We therefore hypothesized that the lipid transport function of CD36 [22] might be linked to the suppressive macrophage phenotype in the TME. To confirm its relevance in human cancers, we observed that CD36 was upregulated in myeloid cells within human NSCLC and melanoma tumors (Fig 1E, 1F) [26, 27]. We also detected significant upregulation of mouse CD36 in tumor-infiltrated vs. splenic CD11b+ myeloid cells in subcutaneous MC38 and B16F10 mouse tumors (Fig 1G), two models characterized as containing lipid-rich interstitium [18]. This supported a conditioning role of the TME on the myeloid compartment. Together, an agnostic phenotypic selection campaign led us to identify CD36 as a candidate target for TAM. We hypothesized that its lipid transport properties were likely a mediator of its pro-tumorigenic properties.

### Functional antibodies bind and block CD36 activity

We next characterized anti-CD36 blocking antibodies in further biochemical and functional screening assays, including our phage-selection derived antibody Macro0168, and two well-characterized, monoclonal antibody clones against CD36. Clone JC63.1 (also known as CRF D-2712) is a mouse IgA blocker of fatty acid and oxLDL uptake with demonstrated anti-tumor activity in preclinical models [33–37]. Clone FA6–152 (mouse IgG1) is a human-specific mouse IgG1 that also blocks TSP-1/2 [38]. Sequences for the JC63.1 and FA6–152 VH and VL regions were obtained by LC-MS-MS analysis of peptide digests, then all three antibodies were produced as both human IgG1-TM effector attenuated and mouse IgG1-D265A effector null formats to reduce FcGR-mediated effector function in macrophage-based assays (Table 1).

**Table 1:** Tool anti-CD36 IgGs.

Sample	Origin	CD36 reactivity
Macro0168 hIgG1TM	Phenotypic selection output	Human
Macro0565 mIgG1 D265A	mIgG variant of Macro0168	Human
Macro0574 mIgG1 D265A	From FA6–152	Human
Macro0568 hIgG1TM	From FA6–152	Human
Macro0579 mIgG1 D265A	From CRF D-2712 (& JC63.1)	Human & mouse
Macro0581 hIgG1TM	From CRF D-2712 (& JC63.1)	Human & mouse

Antibody- and ligand-CD36 association was tested using biolayer interferometry, choosing oxLDL as a specific ligand for CD36 [39]. To measure the direct affinities for human or mouse CD36, the hIgG1TM antibodies were loaded onto anti-human capture sensors with CD36 flowed over. High affinities were observed for hCD36 with the JC63.1 and FA6–152-derived sequences (K_D_ = 9.9 nM and 0.79 nM respectively), while the phage-derived clone Macro0168 had a weaker affinity of 238 nM due to a faster off-rate (Fig 2A, S1 Table). To measure whether the antibodies were able to block the interaction between CD36 and an oxidized lipid ligand, biotinylated recombinant human CD36 extracellular domain was captured on streptavidin, then associated first with the anti-CD36 IgGs or isotype control antibodies, and then with oxLDL. A strong binding signal for oxLDL was observed, which was fully blocked by the JC63.1 and FA6–152 based IgGs, and to a lesser extent by the lower-affinity phage derived IgGs (Figs 2B,C).

**Fig 2:**
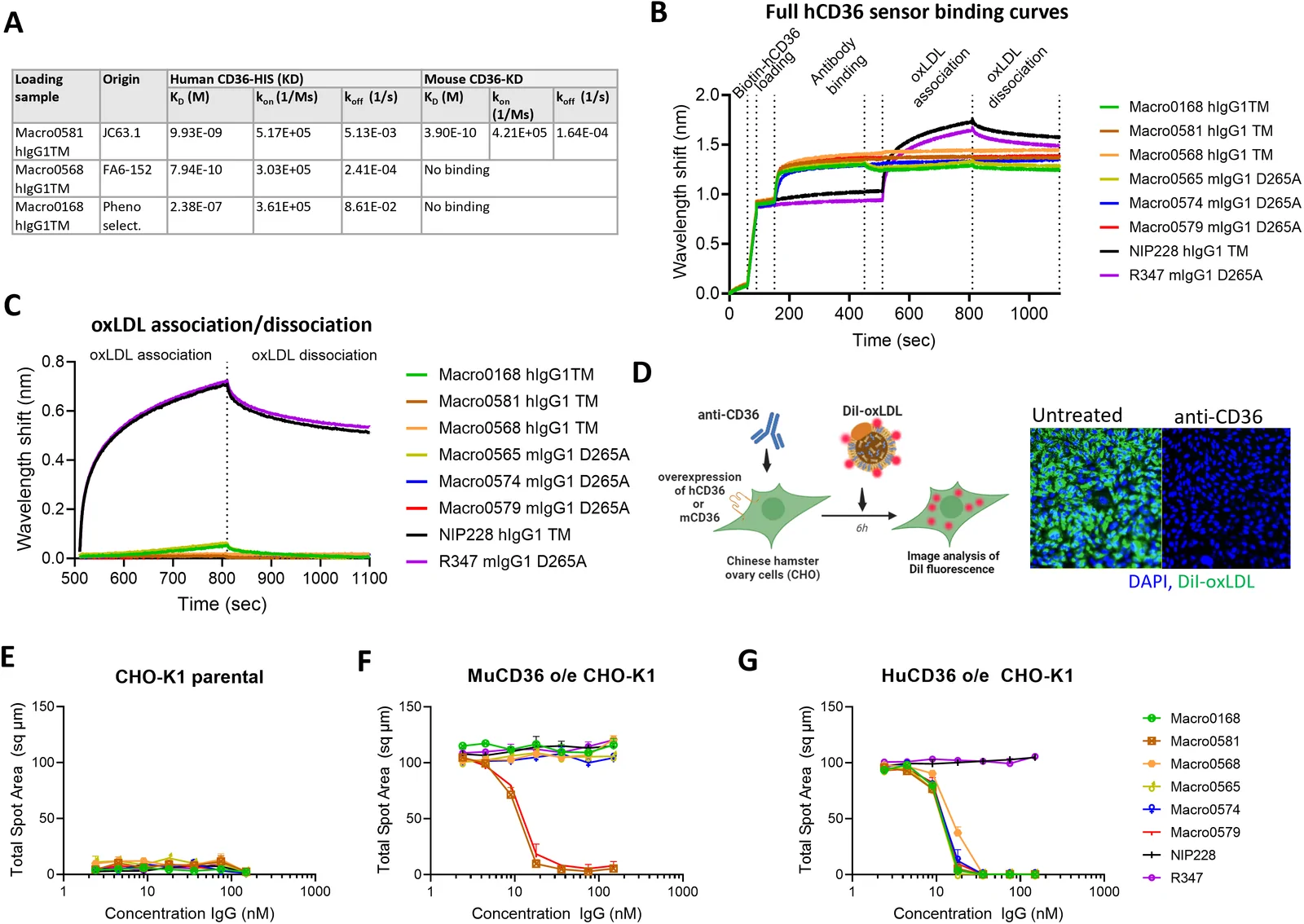
Confirmed CD36 binding and blockade of CD36-mediated lipid transport by anti-CD36 monoclonal antibodies. A. anti-CD36 mAbs from cell selection hits or commercial clones, or isotype controls were captured on anti-human Fc BLI sensors, then sequentially incubated with increasing concentrations of recombinant human or mouse CD36 extracellular domain with a C-terminal avi-tag. Mouse and human CD36 binding affinities and k_on_ / k_off_ rates are shown. In B, profiles are shown for antibodies and subsequently oxLDL binding to immobilized biotinylated human CD36 protein on streptavidin biosensors. BLI sensors loaded with hCD36 were incubated with anti-CD36 antibodies or matched isotype controls, followed by incubation in oxLDL, to test for antibody-mediated competition for the oxLDL binding site on CD36. C. Magnification of oxLDL association/dissociation steps, vertically aligned at the start of oxLDL binding. Immobilized CD36 pre-treated with non-binding mAbs showed binding of soluble oxLDL, whereas CD36 pre-coated with anti-CD36 mAbs blocked oxLDL binding. D. Infographic showing high-content imaging oxLDL cellular uptake assay. CHO-K1 parental cells and cells overexpressing full-length CD36 were incubated for 4 h with 50 μg/mL DiI-oxLDL in the presence of isotype and anti-CD36 IgGs. Representative overexposed 10X-magnified fluorescence images of DiI-oxLDL-incubated cells shows pre-treated or untreated with Macro0579 (50 nM). E-G. Total spot area of DiI-oxLDL in response to titrated IgG1 concentrations in parental CHO (E), mouse-CD36 (F) and (G) human-CD36 overexpressing CHO cells. Minimal uptake is observed in parental CHO cells whereas robust inhibition of uptake is seen in CD36-overexpressing cells blocked with increasing doses of anti-CD36 IgG1.

We next performed high-content imaging lipid uptake assays to assess whether antibody-based inhibition of oxLDL binding to CD36 at a biochemical level corresponded with blocking lipid transport across the cell membrane (Fig 2D). For this we generated CHO-K1 cell lines over-expressing either human or mouse full length CD36 (S1A Fig). Addition of fluorescent DiI-labelled oxLDL to parental CHO-K1 resulted in negligible fluorescence intensity change of cells after 4 h. In contrast, distinct puncta of fluorescent lipid droplets appeared within the cytoplasm in mouse or human CD36-overexpressing cells (S1B Fig), supporting the role of CD36 in lipid transport. Critically, pre-blocking cells with test anti-CD36 IgGs led to dose-dependent reduction in DiI-fluorescence to baseline levels in human CD36 overexpressing cells (Fig 2E,F). Only Macro0579 and Macro0581 (JC63.1 clone) inhibited uptake of DiI-labelled oxLDL into mouse CD36 expressing CHO K1 cells, consistent with its cross-reactivity to human and mouse CD36 protein (Fig 2F).

The oxLDL-binding region on CD36 maps to amino acids 157–171 within a lipophilic binding pocket [40]. We observed that JC63.1 and FA6–152-based clones bind overlapping epitopes on CD36 with Macro0168 in an HTRF-epitope competition assay, despite the differences in mCD36 binding (S1C Fig), and thus are likely to have a similar mechanism for blocking cellular CD36 uptake. We advanced JC63.1 and FA6–152-based clones for further functional testing due to their superior potency over phage selection clones (Fig 2E).

### CD36 blockade is unable to repolarize IL-4 and TCM stimulated human macrophages *in vitro*

We next tested the validated anti-CD36 antibodies for their ability to re-polarize macrophages. In contrast to the engineered CHO cell lines, macrophages express a broad range of scavenger receptors (Fig 3A), granting them redundant mechanisms to recognize altered self or pathogen-associated factors [41]. We used two TAM-relevant models: macrophages differentiated with M-CSF plus tumor-conditioned media (TCM) and IL-4-induced polarization of macrophages. These models generate TAM-like phenotypes in which CD36 has been implicated [14, 32, 42, 43]. Compared to unpolarized cells, TCM-TAM upregulated suppressive markers *MSR1* (CD204), *MRC1* (CD206; mannose receptor) and in some donors, *CD36* (Fig 3C)*.* These changes were also observed at the protein level (S2A Fig). Genes catalysing lipid hydrolysis, *LPL* and *LIPA* (LAL) were also induced on TAM (Fig 3C). However, TAM subjected to 48 h CD36 blockade did not significantly alter *MRC1, MSR1, CXCL10* or *CD80* and therefore did not suggest a re-polarization toward an inflammatory phenotype (Fig 3D), nor did they significantly modulate *LIPA* and *PPARG* – key regulators of macrophage-lipid hydrolysis and metabolism [22, 32] (S3B Fig).

**Fig 3:**
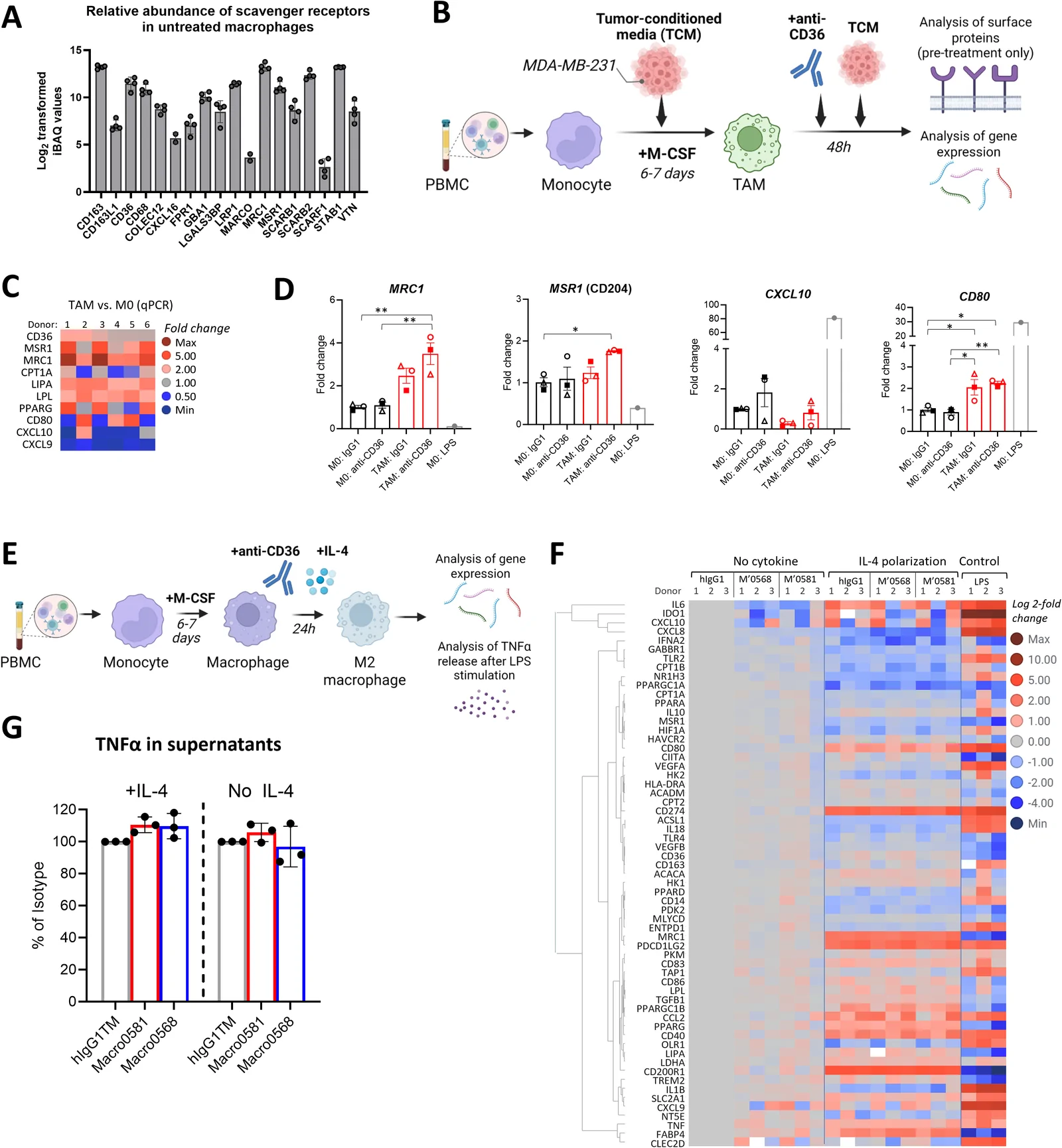
Minimal activity of CD36 blockade in primary human macrophages in TCM-like settings. A. Relative abundance of scavenger receptor expression in monocyte-derived macrophages from four donors. Expression was determined by LC-MS/MS global proteomic analysis. **B.** Schematic showing the derivation of TAM by culturing monocytes with tumor-conditioned media (TCM) derived from MDA-MB-231 TNBC cell line, plus 50 ng/mL M-CSF, and the subsequent biological assessment tested after CD36 blockade. **C.** qPCR assessment of TCM vs. unpolarized ‘M0’ macrophages for a panel of lipid metabolism and macrophage polarization and activation genes in the absence of treatment. Values are expressed as fold-change, with a value of 1 representing no change in expression in comparison to M0. **D.** Expression of M2-macrophage marker genes *MSR1* (CD204) and *MRC1* (CD206), and lipid metabolism genes *CPT1A* and *PPARG*, following 48 h treatment with 7 μg/mL isotype control antibody (mIgG1 D265A) or Macro0579. M0 macrophages stimulated 24 h with 2 ng/mL LPS overnight is a positive control for an activated phenotype*.* Data are reflective of two experimental replicates in macrophages from 6 human donors total. **E.** Schematic showing the derivation of M2-like macrophages from primary human monocytes polarized for 24 with 40 ng/mL IL-4. CD36 antibody blockade was tested during exposure to IL-4. **F.** Macrophages were pre-treated with isotype control (hIgG1TM), Macro0581 or Macro0568 anti-CD36 antibodies during 24 h IL-4 exposure. A panel of genes involved in lipid or glucose metabolism, scavenger receptors and immune modulatory genes was assessed by Fluidigm qPCR. Data is displayed as heat map with hierarchical clustering of genes by negative delta-delta Ct values (log2-fold change). LPS is a positive control for macrophage stimulation. **G.** IL-4-polarized or M0 macrophages were further stimulated another 24 h with 2 ng/mL LPS and TNFα levels measured in cell supernatant by ELISA. TNFα concentrations were normalized by the average technical replicate value from isotype control treated samples. n = 3 human donor values are shown. P > 0.05 by two-way ANOVA (results not statistically significant). Data in **F** and **G** are representative of two independent experiments and 5 donors in total.

To increase the likelihood of capturing expected effects of CD36 blockade on macrophage polarization, activation, lipid transport and metabolism [14, 31, 32, 44], we evaluated these markers using a high-throughput qPCR panel. We observed the expected upregulation of genes post-IL-4 exposure, such as *LIPA*, PD-L1 (*CD274)*, PD-L2 (*PDCDL2)*, *PPARG*, *MRC1*, but little impact of either Macro0568 or Macro0579 treatment within either unstimulated or IL-4 conditions (Fig 3F). Moreover, LPS restimulation of macrophages treated with anti-CD36 did not enhance TNFα secretion over controls (Fig 3G), suggesting cells were not repolarized toward a pro-inflammatory phenotype by anti-CD36 in these systems.

### Minimal activity of CD36 blockade in oxLDL-induced primary macrophages

Minimal CD36 blockade activity in TAM-like *in vitro* assays could be attributed to multiple CD36-independent actions of the TCM and serum lipid constituents, including LCFA, cholesterol and lipoproteins [43, 45]. This might obscure effects attributed to CD36 only. We therefore induced macrophages with oxLDL, a specific ligand for CD36, to potentiate treatment responses. CD36 is the primary transport receptor for oxLDL on macrophages, followed by receptors CD204 and LOX-1 [39, 46]. Pathological oxLDL drives pro-inflammatory signalling, a shift toward glycolytic and reduced FAO metabolic profiles, and a range of phenotypes ranging from M1- to M2-like [47–50]. By differentiating macrophages with oxLDL (Fig 4A), we observed CD36 protein upregulation and suppressive markers CD204, CD163, CD206 (Fig 4B). This effect was more pronounced at higher ligand concentrations. Anti-CD36 blockade did not significantly change expression of these proteins (Fig 4C), nor did it promote TNFα secretion (Fig 4D). CD36 detection was reduced using a competitively binding FACS antibody, indicating that we were achieving high receptor saturation with the blocking antibodies (Fig 4C).

**Fig 4:**
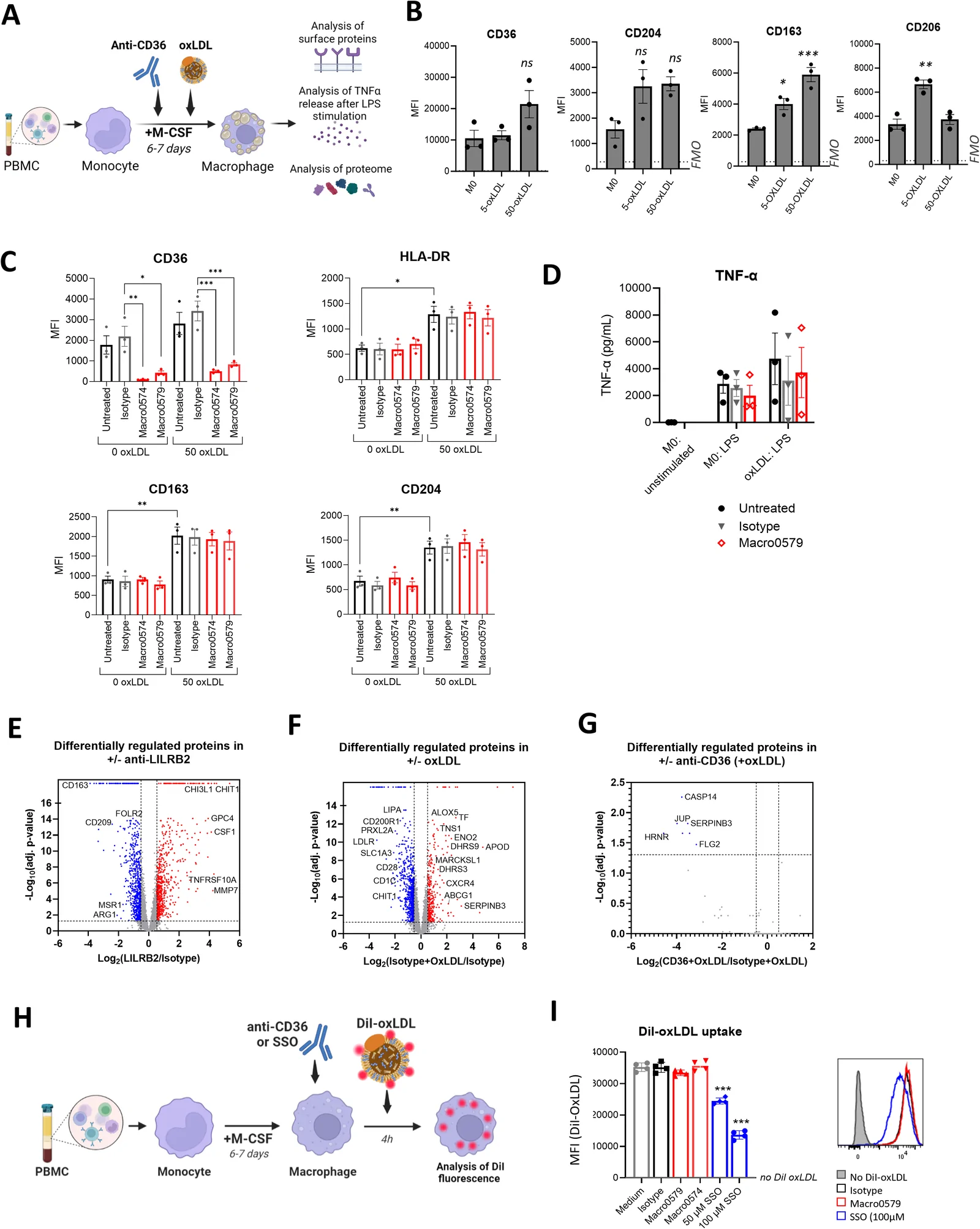
Blockade of CD36 did not significantly alter macrophage response to oxidized LDL. A. Schematic showing how primary human monocytes were treated with M-CSF in the presence or absence of different concentrations of oxLDL for 6 days during their differentiation to macrophages. Antibodies against CD36 were administered throughout conditioning. **B.** The geometric mean fluorescence intensity (MFI) of M2 markers on macrophages from 3 human donors conditioned with 5 μg/mL or 50 μg/mL oxLDL. Data are representative of 4 independent multi-donor experiments. FMO = fluorescence minus one control. ANOVA: CD36: p = 0.0810, CD204 p = 0.0576, CD163 p = 0.0010, CD206 p = 0.0021. Dunnet’s multiple comparisons test results performed on M0 vs. oxLDL concentration. **C.** Surface expression of M2 markers in oxLDL vs. M0 conditioned macrophages treated with 10 μg/mL anti-CD36 antibodies or mIgG1 D265A isotype control. Multiple comparisons tests were performed on 0 vs 50 μg/mL oxLDL, and Isotype vs. untreated or anti-CD36 within each oxLDL condition. ANOVA p < 0.0001 (CD36, CD204, CD163), p = 0.0004 (HLA-DR). **D.** TNFα levels from 3 human donors in the cell culture media supernatants of M0 and oxLDL conditioned macrophages stimulated overnight with 2 ng/mL LPS. Data are representative of two multi-donor experiments. ANOVA test p-value 0.329 (not significant). **E.** Macrophages (4 donors) were exposed to 1 μg/mL Isotype control antibody mIgG1 D265A or Macro0579 + /- 50 μg/mL at monocyte stage and protein extracted from macrophages harvested on day 6. The global proteome was evaluated by LC-MS/MS on protein extracts. Proteins differentially expressed by oxLDL conditioning alone are shown by volcano plot in **F**. Proteins differentially expressed in oxLDL-stimulated macrophages and macrophages subjected to concurrent CD36 blockade vs. isotype control are shown in **G**. Note magnitude of axis scales in G. For comparison, **E** shows significantly modulated proteins following anti-LILRB2 antibody treatment (non-oxLDL). **H.** Schematic of how primary human macrophages were assessed for cellular uptake of DiI-fluorescent oxLDL by flow cytometry following SSO or anti-CD36 treatment. **I.** MFI of DiI-oxLDL on macrophages differentiated from four donors’ monocytes were pre-blocked with 10 μg/mL anti-CD36 antibodies, isotype control or SSO. ANOVA p < 0.0001. By multiple comparisons, significant reductions in DiI fluorescence (***, p < 0.0001), were observed in SSO treatment groups (compared to media), and not (p > 0.05) anti-CD36 treatment groups (compared to isotype). Representative histogram of DiI fluorescence is shown at right. Results are reflective of 2 experimental repeats of 6 donors in total.

We also performed an unbiased global proteomic analysis of the response to anti-CD36 in the same setting. In four human donor macrophages, treatment with our positive control IgG against LILRB2 [51] significantly modulated many proteins compared to control treatment, such as CD163 and CD209 downregulation, as previously described (non-oxLDL condition; Fig 4E). OxLDL conditioning led to significant alterations in many biological processes including antigen presentation, immune responses and metabolism (Fig 4F, S3A and S3B Figs). However, anti-CD36 had negligible activity in oxLDL stimulated conditions (Fig 4G). Together, these data suggested redundant pathway involvement in oxLDL activity on macrophages. Anticipating oxLDL receptor redundancy, we also tested a CD204-blocking antibody in this study (anti-MSR1 H1H21227 [52]), however it too had minimal oxLDL-blocking activity, even in combination with anti-CD36 (S3C and S3D Figs).

We then checked whether the minimal response to anti-CD36 on the proteome level related to the absence of oxLDL uptake by the cells. Notably, we did not observe any significant blockade of DiI-oxLDL uptake in primary human macrophages, even using saturating doses of antibody (Fig 4H, I). In contrast, the CD36 inhibitor sulfo-N-succinimidyl oleate sodium (SSO), potently inhibited DiI-oxLDL fluorescence as previously reported [53]. These results show a discordance between the functional activity of anti-CD36 antibodies between CD36-overexpressing CHO-K1 and macrophages (Fig 2F, 2G).

### Limited anti-tumor activity and antigen sink limit anti-CD36 *in vivo*

We performed parallel *in vivo* studies in mice to test the efficacy and pharmacokinetic properties of the anti-CD36 antibody. Prior reports demonstrated a host-driven oncogenic effect of CD36, evidenced by reduced tumor growth rates in CD36-KO vs WT mice [15, 18, 36, 43]. Although we did not observe this in subcutaneous MC38 tumors [18] (S4A Fig), we found that MCA205 fibrosarcoma tumors grew significantly slower in CD36-KO mice than in WT (Fig 5A). Efficacy was also significantly enhanced by the addition of anti-PD-L1 (Fig 5A), potentially supporting a dual PD-(L)1/CD36 blockade approach to enhance anti-tumor immunity. We then tested whether Macro0579 could offer similar benefit to the global KO. However, dosing Macro0579 at 10 mg/kg IP twice weekly was unable to control MCA205 tumors alone or in combination with anti-PD-L1 (Fig 5B, S4B Fig).

**Fig 5:**
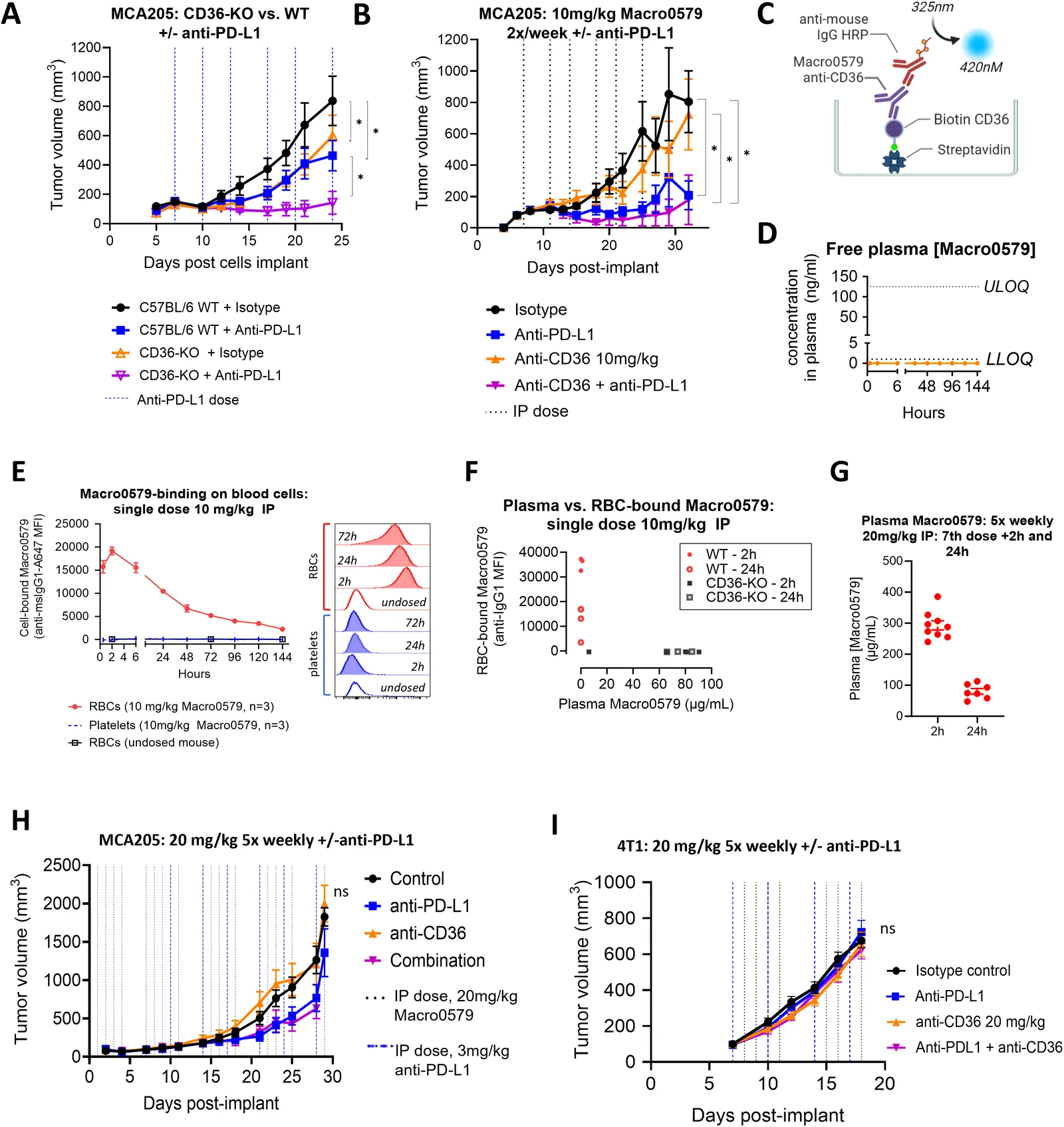
*In vivo* dosing of anti-CD36 antibody results in an antigen sink effect and limited anti-tumor efficacy. **A.** Average volumes of MCA205 tumors grown in WT (C57Bl/6J) and CD36-KO mice. Mice were treated with isotype control antibody or 10 mg/kg anti-PD-L1. Group comparisons were assessed using one-way ANOVA, followed by Fisher’s LSD post hoc tests (*p < 0.05). n = 12 mice/group. **B**. Tumor volume of MCA205 tumors grown in WT mice. Mice were dosed an isotype control antibody or Macro0579 at 10 mg/kg IP with or without anti-PD-L1. Groups were analysed using ordinary one-way ANOVA (p < 0.01) then multiple pairwise comparisons were performed using Bonferroni correction. n = 12 mice/group. **C**. Design of CD36 antigen-capture pharmacokinetic assay for free plasma levels of anti-CD36 antibody. **D.** Macro0579 concentrations from plasma sampled at multiple timepoints (0.5 h to 6 days) after a single 10 mg/kg IP dose of Macro0579 in WT mice. The upper (ULOQ) and lower limits of quantification (LLOQ) of the assay are represented by horizontal dotted lines. **E.** Flow cytometry assessment for red blood cell (RBC)- and platelet-bound Macro0579 in blood cell samples from mice in (**D).** Cellular*-*bound Macro0579 was detected using a fluorescently labelled secondary antibody against mouse IgG1 which was stained *ex vivo.* The mean fluorescence intensity (MFI) was calculated on Ter119 + CD41-CD150- (RBCs) or Ter119-CD41 + CD15 + FSC-low (platelets). **F.** Ter119 + RBC-bound Macro0579 and plasma concentration of Macro0579 in WT C57bl/6J and CD36-KO mice dosed 10 mg/kg (n = 3–5/group). **G.** Plasma concentration of Macro0579 2 h and 24 h after the seventh dose of daily IP dosing (5 days out of 7) at 20 mg/kg administered to WT mice dosed IP. **H.** MCA205 tumor growth curves of C57bl/6J mice treated with 20 mg/kg IP of Macro0579 on the day of cell implant (Day 0). 3 mg/kg anti-PD-L1 was administered starting from day 10 with or without Macro0579. The black line corresponds to control-treated mice. Data show an average of n = 16 mice. **I.** 4T1 mammary fat pad tumor growth curves in Balb/c mice treated with 20 mg/kg Macro0579 IP started 7 days post-tumor cell implant, either alone or in combination with anti-PD-L1. Data show an average of n = 12 mice. The vertical dotted or dashed lines in **A, B, H** and **I** represent dosing days of anti-CD36 (black) or anti-PD-L1 (blue), respectively. *p < 0.05, **p < 0.01.

CD36 has a broad expression profile, expressed for instance on microvascular endothelial cells, adipocytes, and hematopoietic cells such as platelets and erythrocytes [22, 54]. Anti-CD36 is therefore at risk of target-mediated drug disposition (TMDD). Using an in-house developed antigen-capture ELISA (Fig 5C), we measured plasma concentrations of free circulating anti-CD36 from tumor-free C57bl/6J mice given a single dose of Macro0579 (10 mg/kg IP). Macro0579 was undetectable (<LLOQ = 1 ng/μL) at all timepoints evaluated (30 min, 2 h, 6 h, 24 h and up to 6 days post-dose; Fig 5D). Twice-weekly repeat dosing resulted in low levels of plasma Macro0579 at steady state in 6 out of 7 mice (range: 0–26 ng/mL; S4C Fig), together suggesting a large antigen sink might limit the ability of the anti-CD36 antibody to engage with its desired target in the TME causing minimal anti-tumor activity (Fig 5B). Due to the immediate absorption of Macro0579 we observed within the host (<30 min after dosing), we suspected that either platelets or RBCs, cells in high abundance within the circulation, might readily bind any free plasma Macro0579. We assayed blood for cell-bound Macro0579 using an anti-mouse IgG secondary antibody. Although we did not detect Macro0579 on isolated platelets, Macro0579 was strongly bound to RBCs. RBC binding peaked 2 h post-dose and remained detectable after 6 days (Fig 5E). We confirmed target specificity of the sink effect, observing that dosing 10 mg/kg Macro0579 to CD36-KO mice resulted in high levels of anti-CD36 IgG in plasma in the absence of any detection on RBCs (Fig 5F). RBCs were therefore a likely contributor to TMDD of anti-CD36 *in vivo*.

### CD36 blockade dosed to high plasma antibody concentrations is non-efficacious in tumor models

We then developed a modified dosing regimen of Macro0579, finding that 20 mg/kg daily for 5 days a week caused high plasma concentrations at steady-state (2 h-post dose ‘peak’ of 329–385 μg/mL, 24 h ‘trough’ of 48–112 μg/mL; Fig 5G). However, dose-intensive Macro0579 (+/- anti-PD-L1) continued to be inefficacious against established MCA205 tumors (S4D Fig). Administering anti-CD36 at the point of tumor implant, allowing steady-state Macro0579 plasma concentrations to be reached at a smaller tumor volume, was also inefficacious as a treatment strategy (Fig 5H). We tested three further models using the modified dosing to confirm this result. These included two lipid rich models, MC38 and B16, with prior reported efficacy when clone JC63.1 was dosed 10 mg/kg IP every other day [18] (S4E and S4F Figs) and an adipose tissue-rich mammary fat pad implant model 4T1 (Fig 5I). In each case, no significant benefit of CD36 blockade, alone or with PD-L1, was observed. An antibody that competes with Macro0579 binding sites for CD36 (S5A Fig) was used to evaluate receptor occupancy on TAM *in vivo*. Using the B16F10 melanoma model, only modest (~30%) inhibition of Macro0579 binding was observed despite the elevated dose levels and frequency of administration (S5B Fig). Limited efficacy in subcutaneous tumor models is therefore due at least in part to a profound antigen sink in mice and as a result, limited target coverage.

## Discussion

### Summary

Targeting cues that skew macrophage polarization hold promise to reprogram the TME [2], but assessing the druggability of these cues is a necessary step before clinical translation. Having identified anti-CD36 mAbs through a target-agnostic phenotypic screening campaign, we investigated this target further given published evidence linking CD36-mediated lipid transport to immune suppression in macrophages. We found that anti-CD36 blockade, using mAbs able to inhibit CD36-mediated oxLDL uptake in an engineered cell line, did not possess substantial activity in *in vitro* macrophage assays or in *in vivo* primary tumor models. The lack of *in vivo* activity is further explained by two pharmacological liabilities. First, significant TMDD arose from its broad expression profile in tissues and in blood. CD36 is expressed on abundant cells in the circulation such as endothelial cells, platelets, reticulocytes, and mature RBCs [22, 54, 55], leaving negligible free antibody to engage the TME. Second, even at high-dose, high-frequency dosing, target occupancy on tumor-infiltrating myeloid cells reached only ~30% (S5D Fig). Together, these data mean the absence of anti-tumor efficacy in this system cannot be attributed to biological irrelevance of CD36 alone – insufficient pharmacological coverage in the TME remains a critical confound. Addressing both liabilities will likely require next-generation formats. The CD47 field offers a precedent, where RBC-binding and toxicity of first-generation agents were substantially mitigated by bispecific engineering [56]. Whether improved target coverage would translate to efficacy remains uncertain, given the receptor redundancy and functional pleiotropy of CD36 discussed below.

### On receptor redundancy of CD36

Prior reports have implicated CD36 in driving pro-tumorigenic myeloid cells [14, 32, 57], however we could not detect this *in vitro* using IgG1TM-reformatted, commercially available anti-CD36 clones JC63.1 and FA6–152 [33–38]. What was surprising for us was the contrasting profiles of anti-CD36 activity in oxLDL-induced macrophages (near-absent responses) vs. CD36-overexpressing CHO cells that do not normally express scavenger receptors (strong responses). This discrepancy may be explained by the redundant mechanisms by which macrophages acquire lipids, which include most scavenger receptors, TLR4, phagocytosis of cells, non-receptor mediated pinocytosis and multiple fatty acid transporters such as FATP1, FATP2, FATP5, FABP [49, 58]. In CD36-deficient patients, monocyte-derived macrophages took up only 40% less oxLDL than non-deficient patients, meaning 60% of the lipid uptake was attributable to redundant pathways [59]. Dual CD204/CD36 blockade was also inactive in our studies, so it appears that additional mechanisms beyond these scavenger receptors may dilute the overall contribution of CD36 to lipid uptake processes in macrophages.

### On pleiotropic functions of CD36

Given extensive literature on CD36-mediated lipid uptake in macrophages, the absence of efficacy of anti-CD36 was striking. However, our review of the literature shows that published CD36-dependent macrophage phenotypes are primarily described in settings of complete gene disruption such as in CD36-deficient patients [59], CD36 global-KO mice [14, 15, 60] and myeloid-specific conditional KO mice [57]. SSO is also commonly used to pharmacologically block CD36 [14, 32, 53, 61], however it is not specific for membrane CD36 and interacts with respiratory chain enzymes [62]. We found few examples of using CD36 antibody blockade to modulate macrophages *in vitro*, with those that do showing effect sizes that range from absent-to-modest [48, 63–65] or require simultaneous LPS-TLR2/4 stimulation [48, 66]. CD36 has dozens of ligands, with different binding sites reported [21]. CD36 also interacts with membrane receptors TLR2/4/6, integrins, tetraspanins and NKA in response to PAMPs and DAMPs, which can trigger multiple signalling pathways including MAPK, AMPK, NFKB and SFKs [22]. CD36 in TAMs has further been reported to suppress type-I IFN signalling through p38 activation downstream of oxidized lipid signalling, thereby dampening anti-tumor immune responses [57]. This pleiotropy means that the KO of CD36 gene will alter multiple functions of macrophages and will thus likely overestimate what can be achieved through blockade of one specific CD36 function.

### On phenotypic selections on TAM

Phenotypic screening offers a first-in-class opportunity for novel target discovery [12] but is a long, resource-intensive process that is prone to generating false-positives [67]. One limitation is that the success of screen outputs is dependent on the design and triage strategy of the disease-relevant screening model [67]. Human TAM are notoriously complex, heterogeneous, and plastic [8, 13], are difficult to model *in vitro*, and access to primary samples is limited. We therefore undertook phage selections applying a classic M1/M2 polarization protocol of IFN-γ and IL-4, a common practice at the time the screen was undertaken. We also applied LPS, a byproduct of gram-negative bacteria and potent stimulator of TLR2/4, to improve the assay window as part of the requirements for feasibility of a large scale, high throughput screen. As a result, the antibodies were screened for their ability to enhance the activated state of human macrophages, rather than reverse their immunosuppressive state. Ultimately, cell-binding IgG hits were not M2-selective. CD36 also co-operates with TLR2/4/6 receptors and thus is involved in innate signalling and lipid sensing [68]. In retrospect, these stimulation conditions might have inadvertently enriched for homeostatic and TLR-driven innate signalling co-receptors in the phenotypic selection model. Until suitable and scalable models that recapitulate diverse TAM populations become available, phenotypic selections in this cell population may remain a challenge. A final limitation of phenotypic selection is that the knowledge of the target’s mechanism of action during screening may not be known. Developing new functional screening assays off the back of the selection model can be challenging if the MOA is unknown or complex [12], as is the case with CD36. Notably, we ceased to find anti-CD36 activity outside of phenotypic selection screens, where we focussed on an assumed lipid transport function.

Despite CD36’s negative outcome as an antibody target, the workflow described here – combining target-agnostic phage selection on primary human macrophages, high-content imaging, high-throughput functional screening, arrayed cDNA library-based target deconvolution, and comprehensive in vitro and in vivo validation – provides a broadly applicable framework for identifying and rigorously evaluating therapeutic candidates in tumor-associated macrophages. By identifying CD36 and subsequently demonstrating its unsuitability under the conditions tested, this approach highlights the value of systematic de-risking and is readily transferable to other immune-oncology targets and cell types.

## Conclusions

In conclusion, we caution those in pursuit of targeting CD36 through antibody blockade, including in non-oncology indications such as atherosclerosis where macrophage oxLDL-CD36 is considered a pathological driver of plaque formation [50]. Broad expression and diverse functional roles for CD36 may limit efficacy of this approach, particularly when targeting macrophages. Importantly, the limitations we describe regarding systemic antibody blockade of CD36, including incomplete target engagement in tumor-infiltrating myeloid cells, should not be interpreted as evidence against a biological role for CD36 or lipid metabolism in suppressive TAM, but rather that alternative pharmacologic approaches might be required to achieve adequate target coverage in the TME. Our findings may not necessarily translate to non-myeloid-based oncology strategies that block CD36 for metabolic reprogramming of T-cells [18, 37], or metastatic tumor cells [35]. Notably, a recent report documented profound anti-tumor efficacy in genetically engineered mouse models of hepatocellular carcinoma but limited activity in subcutaneous tumor models using a novel CD36 blocking antibody targeting the lipid binding pocket of CD36 [69], supporting the view that CD36 lipid-sensing function and signalling pathways play a key role in tumor progression in certain contexts [35, 69]. Nonetheless, classical monoclonal antibody approaches would be subject to the TMDD we observed with anti-CD36 mAbs and require mitigation for further development.

## Supporting information

S1 Fig Specificity of anti-CD36 binding in biochemical and engineered cell lines. **A.** CD36 expression profiles on engineered CHO-K1 CD36 overexpressing cell lines as detected using flow cytometry. Cells were utilized in an image-based oxLDL uptake assay. **B.** High content imaging of DiI-oxLDL treated cells co-stained for nuclei with DAPI (blue) and either untreated or treated with 150 nM Macro0579 in CHO-K1 parental, or mouse/human CD36 overexpressing cells. The inset demonstrates intracellular/peri-nuclear DiI fluorescence. Scale bar represents 200 μm. **C.** CD36-Macro0168 epitope binding HTRF competition assay. Macro0168 was labelled with Dy650 and competed with test IgGs for their ability to bind CD36. Complete inhibition of signal by anti-CD36 IgG co-incubation indicates epitope competition by these mAbs. Left-shifting curves correspond to higher-affinity binders for CD36.
(TIF)

S2 Fig Minimal impact of CD36 blockade on oxLDL uptake and polarization of primary human macrophages. A. Primary human monocytes were isolated and conditioned with or without tumor-conditioned medium (TCM) and profiled by flow cytometry. The geometric mean fluorescence intensity (MFI) of M2 surface markers in macrophages from three human donors. FMO = fluorescence minus one control. P-values for a paired t-test are indicated, where ns = not-significant, p > 0.05, *p < 0.05. **B.** Expression of lipid metabolism genes *PPARG* and *LIPA* as assessed by qPCR, following 48 h treatment with 7 μg/mL isotype control antibody (mIgG1 D265A) or Macro0579. M0 macrophages stimulated 24 h with 2 ng/mL LPS overnight is a positive control for an activated phenotype*.* Data are reflective of two experimental replicates in macrophages from 6 human donors in total. ANOVA p = 0.0367 (*PPARG)* and p = 0.0578 (*LIPA).* Dunnet’s multiple comparisons p-value as indicated represent p < 0.05 (*), p < 0.01 (**) or p < 0.001 (***).
(TIF)

S3 Fig Proteomic changes of primary human oxLDL-conditioned vs M0 macrophages. **A, B.** Monocytes from 4 human donors were exposed to +/-50 μg/mL oxLDL throughout conditioning to macrophages. The global proteome was assessed by LC-MS/MS. Monocytes were isolated and treated for 6 days with 1 μg/mL Isotype control antibody mIgG1 D265A, Macro0579, anti-CD204 IgG1 TM or the combination of Macro0569 + anti-CD204 in the presence or absence of 50 μg/mL. **A.** Pathways significantly enriched and (**B**) significantly downregulated by oxLDL treatment (in the absence of antibody treatment). **C, D.** Proteins differentially expressed in anti-CD204 vs. isotype control (**C)** or the combination of anti-CD204 + anti-CD36 vs. isotype control (**D**) in the presence of oxLDL are shown by volcano plot.
(TIF)

S4 Fig Anti-CD36 antigen sink and anti-tumor inefficacy *in vivo.* **A.** Average MCA205 tumor volumes in WT and CD36-KO mice, with mice treated with or without 10 mg/kg anti-PD-L1. N = 12 mice/group. Group comparisons were conducted using non-parametric one-way ANOVA (Kruskal–Wallis test), followed by Dunn’s multiple comparison test for pairwise comparisons. (*p < 0.05). **B.** Individual tumor growth volumes for MCA205 tumors treated with isotype control or 10 mg/kg anti-CD36 (Macro0579) twice a week for 4 weeks. **C.** Plasma concentrations of Macro0579 from mice bearing MCA205 subcutaneous tumors that were administered 10 mg/kg IP of Macro0579 twice weekly. Plasma was isolated from blood taken from mice 1 h after the 6^th^ dose to acquire a steady-state sample. Top panel: Study schematic. **D.** Average tumor growth curves for MCA205 tumors treated with 20 mg/kg of anti-CD36 (Macro0579). Treatment began 7 days after tumor cell implant. 3 mg/mL anti-PD-L1 was administered starting from day 10 in combination with or without Macro0579. N = 11 (control), 13 mice (anti-PD-L1), 12 (Macro0579) or 15 mice (combination). Top panel: Study schematic. **E.** Average tumor growth curves for MC38 tumors treated with 20 mg/kg of Macro0579 7 days after tumor cell implant. 10 mg/mL anti-PD-L1 was administered starting from day 7 in combination with or without anti-CD36. Black line corresponds to control-treated mice. N = 10/group. **F.** Tumor growth curves for B16F10 tumors treated with 20 mg/kg of Macro0579, 7 days after tumor cell implant. 10 mg/mL anti-PD-L1 was administered starting from day 7 in combination with or without Macro0579. N = 14 mice per group. The vertical dotted or dashed lines in all panels represent dosing days of Macro0579 (black) or anti-PD-L1 (blue), respectively. In **A, D, E,** and **F** a Mann-Whitney U test was performed to measure statistically significant differences in growth rates compared to isotype control treatment group. p < 0.05 (*), p < 0.01 (**) or p < 0.001 (***).
(TIF)

S5 Fig Target engagement of Macro0579 *in vivo.* A. An antibody competition assay designed to identify a non-competing clone against CD36 to evaluate target engagement by Macro0579. 1 nM of fluorescent dye-labelled anti-murine CD36 antibody clone HM36 was co-incubated for 3 h on ice with a titration of unlabelled test antibodies. B. Results of competition assay in which loss of fluorescence indicates competition for CD36 binding with HM36. C. Schematic of a study to evaluate CD36 target engagement in tumors in the B16F10 melanoma model. D. Tumor samples derived from were dissociated 24 h post-final IP dose of Macro0579 20 mg/kg and stained for myeloid cell markers and CD36. Partial CD36 engagement by Macro0579 is observed in anti-CD36 treated groups (n = 4–9/group). MFI = mean fluorescence intensity.
(TIF)

S1 Table Kinetic values of tool anti-CD36 mAbs. (XLSX)

S2 Table Flow cytometry reagents. (XLSX)

S3 Table Taqman qPCR reagents for gene expression assays. (XLSX)

S4 Table Data from Fig 1. (XLSX)

S5 Table Data from Fig 2. (XLSX)

S6 Table Data from Fig 3. (XLSX)

S7 Table Data from Fig 4. (XLSX)

S8 Table Data from Fig 5. (XLSX)
